# The Complexities of AI for Social Good: An Explorative Study on Adversarial Machine Learning to Facilitate Deliberative Decision-Making in Elections

**DOI:** 10.1007/s11948-026-00598-9

**Published:** 2026-04-30

**Authors:** Syafira Fitri Auliya, Olya Kudina, Aaron Yi Ding, Ibo Van de Poel

**Affiliations:** 1 Section Ethics and Philosophy of Technology, Department of Values, Technology, and Innovation, Faculty of Technology, Policy, and Management, TU Delft, Jaffalaan 5, Delft, 2628 BX The Netherlands; 2 Department of Engineering Systems and Services, Faculty of Technology, Policy, and Management, TU Delft, Jaffalaan 5, Delft, 2628 BX The Netherlands

**Keywords:** Deliberative decision-making, Elections, Privacy, Adversarial machine learning, Artificial intelligence, Micro-targeting political campaigns

## Abstract

The proliferation and pervasive use of artificial intelligence (AI) pose significant challenges to our democracies. In particular, AI leverages microtargeting political campaigns by constructing detailed user profiles and inferring people’s individual susceptibilities from their data. This capability enables highly targeted political messaging that can substantially influence voting decisions, potentially undermining citizens’ ability to make deliberative decisions in elections. While existing research has proposed interventions to mitigate the negative impacts of political micro-targeting, many of these interventions may become less effective as AI continually yields more powerful and subliminal forms of micro-targeting. Assuming that technologies can play a role in mitigating these negative impacts of AI, we conducted an explorative study to identify the design principles of technological tools that could facilitate citizens’ deliberative decision-making in elections in the continuously levelaged AI-based political campaigns. Using Indonesia’s 2024 elections as a case study, we interviewed twenty citizens and four political actors to gain critical insights into how such technologies might be developed. Initially, we anticipated that privacy-enhancing AI, used to counterattack profiling AI used by political actors, might suffice to facilitate election deliberation. However, our findings reveal a more complex reality: addressing societal issues through technology is inherently challenging; no single solution can serve as a silver bullet. Instead, facilitating election deliberation requires integrating privacy with other conditions, including self-reflection, education, access to diverse information, critical thinking, and openness to others. These design principles might serve as concrete, actionable design principles to guide the development of technologies to enhance election deliberation.

## Introduction

The advancement of AI has spurred debate surrounding its societal impacts, particularly its capacity to undermine democratic institutions (Manheim & Kaplan, 2019; Brkan, 2019; Helbing et al., 2019; Ünver, 2018; Zittrain, 2014). A prominent example is the Cambridge Analytica scandal, which demonstrated how the misuse of AI could pose risks to the electoral system. This scandal drew attention to the critical contributions of AI in at least two domains: analyzing vast datasets obtained from social media platforms and enabling targeted election campaigns that shifted from traditional generic to more micro-targeted influences (Cadwalladr & Graham-Harrison, 2018; Rosenberg et al., 2018). With political actors trying to drive people’s political choices by continuously providing personalized political campaigns based on information gathered from their constituents, this AI-aided campaign strategy poses a significant threat to democracy. One of the pillars of democracy is the citizens’ equal participation in the decision-making processes (Dahl, 2020; Elster, 1998), which requires citizens to have autonomy in their political participation and the capacity to make deliberative, reflective decisions. This means that the fundamental principle of democracy is compromised when individuals’ ability to make deliberative decisions is disrupted by powerful personalized influences that attempt to direct people’s political decisions and bypass the reflectivity they should have before making these decisions.

The global reaction to the Cambridge Analytica case was significant, prompting social media platforms, including Facebook, to revise their data handling policies to prevent political profiling^1^. Despite these efforts, the potential for misuse of social data for political profiling remains, as demonstrated by recent research, still showing the high accuracy with which social media data can predict political preferences (Baran et al., 2022; Belcastro et al., 2020; Campanale & Caldarola, 2018; Kitchener et al., 2022). This vulnerability underscores the need for mechanisms that enable citizens to have a stake in resisting political profiling and preserving the deliberative nature of their political choices.

To counteract such possibilities, the potential application of Adversarial Machine Learning (AML) is worth exploring. AML, which uses an AI to create small non-random perturbations to change the output of another AI’s system (Szegedy et al., 2014), offers a promising avenue for mitigating the risk associated with some AI applications (P.-Y. Chen, 2022). Some researchers have proven that applying AML can tackle some of AI’s negative social impacts, such as violations of copyright and privacy (P.-Y. Chen, 2022; Sablayrolles et al., 2020; Shan et al., 2020, 2023, 2024). AML may also be used to counterattack targeted micro-targeted campaigns based on AI-driven political profilers by using AML to obscure social media data, thereby impairing the accuracy of AI systems in profiling users’ political preferences (Auliya et al., 2024) 

In this paper, we are interested in whether an application of AML can enhance people’s election deliberation based on stakeholders’ perspectives. In this, we use the methodology of Value Sensitive Design (VSD), a methodology that proactively and reflectively integrates specified stakeholder values in the design process (Friedman et al., 2006), especially focusing on its empirical stage. While 2024 was marked by many presidential elections across the world, the one in Indonesia was one of the earliest in the year and where one of us (SFA) had meaningful societal embedding. For these reasons, we focused on the case of elections in Indonesia and carried out a series of semi-structured interviews with a diverse group of respondents, including citizens, politicians, campaign teams, and consultants in Indonesia during the 2024 election period. With this fieldwork, our goal was to empirically underpin the exploration of the following research questions: *What are the possible design principles of the tools to facilitate deliberative decision-making in elections?* By integrating feedback from these interviews, we endeavor to sharpen the tool’s design, ensuring that it aligns with the needs and expectations of those it seeks to empower.

With the goal of facilitating individuals’ deliberative decision-making in elections, this research is an explorative empirical study to investigate the design principles of technological solutions sharpened by the theoretical framework and empirical study. Section  “Theoretical Background” delves into the overall methodology of VSD and design principles, as well as the theoretical framework of the risk of personalized political influences and the concept of AML for good purposes. Section  “Research Approach” shows the methods employed in this study. Section  “Results” details the findings. Section  “Discussion” discusses the implications of the findings for achieving the goal of facilitating election deliberation, provides practical recommendations, and outlines the limitations of this study. The conclusion is provided in Section  “Conclusion”.

## Theoretical Background

### Value Sensitive Design and Design Principles

VSD is a design approach aimed at embedding values of moral importance throughout the design processes. It proactively considers the implications of the technological and design choices (Friedman et al., 2006). By taking these implications into account, designers can anticipate both direct and indirect implications of the technologies that they develop, as well as embed values during the design process (Verbeek, 2009).

VSD involves three interconnected phases: conceptual, empirical, and technical investigations. During the conceptual phase, VSD identifies the values impacted by the technology and handles the trade-offs among competing values. The empirical phase investigates human activities associated with the designed technology. It involves examining stakeholders’ understandings, contexts, and experiences of the technologies and their implicated values (Friedman & Kahn, 2003). The technical investigation involves designing systems that fulfill the values identified in the conceptual phase, including thoroughly examining existing related technological solutions.

Building on this approach, a conceptual investigation was conducted by Auliya et al. (2024) to explore democracy, privacy, and the interrelations of these values. In this paper, we expand on their conceptualization and focus on the empirical investigation. Specifically, we explore stakeholders’ perceptions regarding potential technological solutions, highlighting the intersection between deliberative decision-making in elections, privacy on social media, and AML. All of these uncover the values most relevant to stakeholders, which are then translated into design principles of the technologies proposed.

To translate values into design principles within VSD, we adopt the value hierarchy proposed by Van De Poel (2013). This hierarchical structure organizes values, norms, and design requirements, where values are the highest level, specified through norms and design requirements. While Van de Poel uses the term “design requirement” for the most detailed specifications, we refer to these as “design principles” to avoid ambiguity with the design requirements commonly understood in computer science, which refer to more detailed and technical criteria. The elements in the lower levels are done for the sake of the higher-level elements. This translation of values into design principles helps lay the groundwork for future technical investigations. By iterating between design principles and technical investigations, designers can refine their decisions and ensure that the technology built remains value-driven in all design processes.

VSD was originally developed in the Human-Computer Interaction (HCI) community (Friedman et al., 2002). Within HCI and software engineering more broadly, VSD coexists with a range of other human-centered and user-centered design approaches. For example, Personas methods have lately been extended to the design of AI systems. It has been used to map users’ mental models in the development of human-centered AI applications (Holzinger et al., 2022). In software engineering, user-centered software development has also emphasized the importance of integrating user needs and stakeholder considerations throughout the software development lifecycle, particularly from its early stages (Peischl et al., 2015). All these approaches are not mutually exclusive, but can be complementary, depending on the design goals and the stage of development. For instance, Personas can support VSD by concretizing stakeholders and reflecting users’ mental models, therefore enriching VSD’s empirical investigation; while user-centered software development helps clarify how such value-oriented insight can be operationalized in concrete development processes. The integration of VSD with AI-based technologies has also been articulated, extending VSD to the distinctive attributes of AI systems (Sadek et al., 2024; Umbrello & van de Poel, 2021).

### The Risk of Personalized Political Influences

Personalized political influences, often also called ‘micro-targeting political campaigns,’ allow political actors to provide tailor-made political campaigns to each user. So that different users might receive different campaign materials. We define micro-targeting political campaigns in a nuanced way as the political campaign strategy that selectively targets specific individuals based on their Personal Identifiable Information (PII) (such as residential address and phone number) as well as the strategies that target groups of individuals based on shared characteristics (such as religion and postcode) to provide the targeted and personalized campaigns materials.

Scholars have long contested the effectiveness of micro-targeting political campaigns in impacting election outcomes, especially compared to traditional campaigning. However, there is increasing evidence of the dangers of political micro-targeting. This is not just based on the story of the Cambridge Analytica in 2016; researchers also highlighted comparable research findings. In 2018, Hazenberg et al. showed that simple demographic characteristics could be used to predict the voting behaviors of increasingly smaller groups of citizens. They mentioned that a Dutch foundation, hired by over 70 political parties, combined poll results with other data, such as municipal data, to identify party supporters’ locations and potential supporters. This allowed them to refine the aggregated information from 1,200 to 35 individuals, enhancing the precision of targeted campaigns to better align with the characteristics of the intended smaller groups (Bouwman, 2018; Hazenberg et al., 2018). In May 2023, the US Senate Hearing on AI questioned the OpenAI CEO and other experts over the use of Large Language Machines (LLM) to forecast public opinion and manipulate specific behavioral responses, particularly during elections and with indecisive voters. They also raised concerns about the vast amount of data gathered by large platforms to the point where they “know of us better than ourselves.” (*OpenAI CEO Testifies on Artificial Intelligence*, 2023). All of these showed that, in this digital era, greater access to detailed data and enhanced capacity of technologies enable political actors to more accurately predict voters’ political preferences, improving the precision of micro-targeting political campaigns.

However, a recent white paper from the University of Chicago and Stanford Graduate School of Business challenges the impact of political micro-targeting in the 2024 US elections. They believe that the efficacy of these campaign strategies is questionable. According to their arguments, although AI has the capability to automate the production of campaign materials that are nearly identical to those created by humans, its application in mass persuasion and voter manipulation is improbable due to the high cost of implementing generative AI, the risk of retaliation from political opponents or the media, and its potential to cast doubt on the legitimacy of the candidate’s victory (Mesquita et al., 2023).

Although Mesquita et al.‘s doubts about micro-targeting political campaigns’ efficacy might have merit in a particular context, we would argue that their relevance would diminish in different sub-ideal political environments. For instance, in a corrupted political setting where a candidate has control over communication channels and government institutions, the candidate might be less concerned about encountering counter-information and backlash from their opponent. Moreover, if the candidate has control over the institutions that validate election legitimacy, they will feel confident about their win’s legitimacy even if an official trial is launched to examine the win achieved through micro-targeting campaigns. On top of that, the public’s limited awareness of the detrimental effects of tailored political influences will also significantly influence the reception of AI-supported campaigns. Under these circumstances, personalized political campaigns could potentially arise and negatively affect democratic practices.

Indeed, the exact impact of micro-targeting political campaigns on voters’ decisions and election outcomes is hard to determine. Voters’ decisions are determined by a multitude of factors that are interconnected. Nevertheless, recent studies have simulated the effectiveness of this approach in persuading voters. In 2024, a study involving over one thousand participants demonstrated that personalized political advertisements that were customized to individual personalities were more effective than non-personalized ads. To also prove that the recently accessible LLMs are able to create these personalized political advertisements, they prompted GPT-3 and ChatGPT to rephrase the existing ads, tailoring the advertisements to people with different characteristics (Simchon et al., 2024). A larger-scale study with over twenty thousand participants was also conducted by researchers from MIT and Stanford. They found that their microtargeting strategy has an average of 70% larger persuasive impact than other non-personalized messaging strategies (Tappin et al., 2023). While both studies emphasize that the effectiveness of micro-targeting political campaigns is context-dependent, they also suggested the potential impact of these campaigns in reshaping democracy compared to traditional campaign strategies.

Our broad definition of democracy is “a method of collective decision-making characterized by a kind of equality among the participants at an essential stage of the decision-making process” (Christiano & Bajaj, 2022), with each participant “having equal and effective opportunities for learning about the relevant alternative policies and their likely consequences” (Dahl, 2020). In addition, the legitimacy of a democratic society, especially in a modern democracy, comes from the discussion and general deliberation that precede voting (Chambers, 2012; Goodin, 2008; Manin, 1987). Therefore, political micro-targeting campaigns may undermine democratic values by impeding people’s access to alternative and nuanced perspectives on topics beyond those presented to them through the campaigns. Individuals could be exposed to personalized campaigns of varying degrees, contingent upon the quantity and nature of data collected about them. Consequently, their opportunities to engage in discourse prior to voting would be diminished to varying degrees.

In addition, micro-targeting political campaigns often violate privacy. Especially if it includes inferring sensitive information not disclosed by users, which can be generated by combining non-personal data, aggregated, and anonymized data (Auliya et al., 2021; Hazenberg et al., 2018). When this information extends beyond what users willingly disclose, it contradicts the definition of privacy as “the right to be left alone”, where people should be protected against unwanted exposure and scrutiny (Warren & Louis, Brandeis, 1989). Moreover, giving people tailored messages to influence them is also another form of privacy violation, where people are “forced to hear propaganda, [be] manipulated by subliminal advertisements, or [be] disrupted by a nuisance that thwarts one’s ability to think or read” (DeCew, 1997; Solove, 2002).

Scholars have long proposed solutions to address these challenges by linking the negative impacts of political micro-targeting campaigns of decreasing access to diverse perspectives and giving external stakeholders the power to stir people’s election decision-making. For example, Bozdag and Van Den Hoven gave an overview of tools to expose users to diverse viewpoints, enabling them to discover disagreements, check the facts, and align perspectives to facilitate better decisions. These tools allow users to visualize and discover different viewpoints, present pros and cons arguments for a given topic, nudge users to listen more attentively, and modify users’ political search queries to diversify search results (Bozdag & Van Den Hoven, 2015). However, while most of these tools have undergone empirical testing, the study itself was published in 2015, making these tools potentially less relevant in future possibilities where AI may lead to more powerful, subliminal, and pervasive political micro-targeting campaigns, as well as the increased penetration of social media. Meanwhile, while Auliya et al. (2024) envisioning the future development of AI by proposing the idea of using AI to counterattack profiler AI used in political micro-targeting campaigns, their study is still only on a conceptual level that neither has empirically explored nor detailed into any design principles. Therefore, this paper aims to fill this gap by exploring such design requirements using empirical means.

### Adversarial Machine Learning for Benefiting Society

AML is a growing field of computer science that exploits the characteristics of deep neural networks, in which non-random alterations in the data input can change the network’s prediction without being identified by the system (Szegedy et al., 2014). In other words, it makes AI systems to produce incorrect results as a result of intentionally providing them with deceptive input. AML was initially perceived as undesirable because it can impact the model’s training data and change the outcome (Usynin et al., 2021). However, as AI advances and human cognitive abilities struggle to keep pace, a new perspective is emerging on AML. AML may also be used for good purposes by counterattacking malicious AI (P.-Y. Chen, 2022).

There are several widely recognized cases where AML aided people in mitigating social risks presented by AI systems. In 2023, a tool called *Glaze* was launched to counter AI models that use artists’ work for training without consent and even imitate their art style. Using *Glaze*, artists can put a ‘poison’, operating at the pixel level unnoticeable by human eyes, in their images to obscure them and prevent text-to-image diffusion model tools from mimicking their styles (Shan et al., 2023). Additionally, the same research team also created *Nightshade*, a tool designed to impair AI models that ignore the do-no-crawl directive from the image owners (Shan et al., 2024). Sablayrolles et al. also implemented watermarking on images to facilitate dataset tracking when the dataset is employed as a training model. Even with just 1% of the watermarked dataset utilized, it is still possible to trace the dataset (Sablayrolles et al., 2020). Another strategy is put forth by Shan et al., which involves introducing subtle pixel-level modifications to a user’s photos in order to avoid user-recognition devices. Their research demonstrated that this cloak offered over 95% protection against user-recognition services (Shan et al., 2020). The aforementioned developments show that AML may not only be about adversarial attacks with negative consequences but may also be potentially used for good purposes.

## Research Approach

We conducted two interview series. The first series, which involved twenty citizens, explored the perspective of citizens whose election decisions may be impacted by micro-targeting political campaigns. The second series involved four political actors, with the goal of exploring the strategies they employed during the 2024 Indonesian elections, especially those that involved micro-targeting strategies.

The semi-structured method was used as a data-gathering strategy to investigate the respondents’ viewpoints, due to its adaptability in conducting probing conversations and its flexibility to pursue a line of discussion (Edwards & Holland, 2013). This method is considered suitable for this research since the participants have a wide range of knowledge and personal backgrounds, requiring different approaches to provide essential insights. For selecting respondents, we used the purposive sampling strategy to find information-rich respondents who can provide substantial insights into the research queries. More specifically, in selecting information-rich cases in the purposive sampling strategy, the maximum variation sampling approach was used to capture common patterns and ensure that demographical variation is reflected in the limited-sample qualitative study (Patton, 2000). This approach suits Indonesia’s diversity which might influence voting behavior.

We chose to study Indonesia’s 2024 election since it was one of the earliest in the year, and one of us had meaningful societal embedding in there. Twitter was the specific main context of our study, as many recent empirical studies demonstrated that Twitter data could be used to reveal the political preferences of individuals (Baran et al., 2022; Belcastro et al., 2020; Campanale & Caldarola, 2018; Kitchener et al., 2022), which could be leveraged in political micro-targeting campaigns. However, one limitation of our study is the limited number of Twitter users compared to other leading social media platforms in Indonesia, which puts a constraint on the scope of our research.

To study citizens’ perceptions, twenty Indonesians were interviewed. Following our maximum variation sampling approach, we selected respondents with varied education levels, residences, ages, social media usage, and professional backgrounds to gather a common understanding from a variety of perspectives. All of the respondents were eligible to vote in the Indonesia 2024 elections. We initiated the semi-structured interview by demonstrating how AI may use their data to provide personalized influences that impact their daily decisions, such as changing their preference from buying one brand of bags to another. We then associated AI’s ability to impact individuals’ choices with events in some countries’ elections, illustrated by the Cambridge Analytica scandal. We explained that personalized influences contradict Indonesia’s well-known election principles, *‘Luber Jurdil*,’ particularly in the *‘ber’* (freedom), which encompasses the necessity for citizens to engage in deliberative decision-making and not be stirred by external influences. We then queried them on their social media usage, factors affecting their election decision-making, and the impact of instances of social media on changing their election choices. A more detailed list of questions is available in the Appendix.

**Fig. 1:**
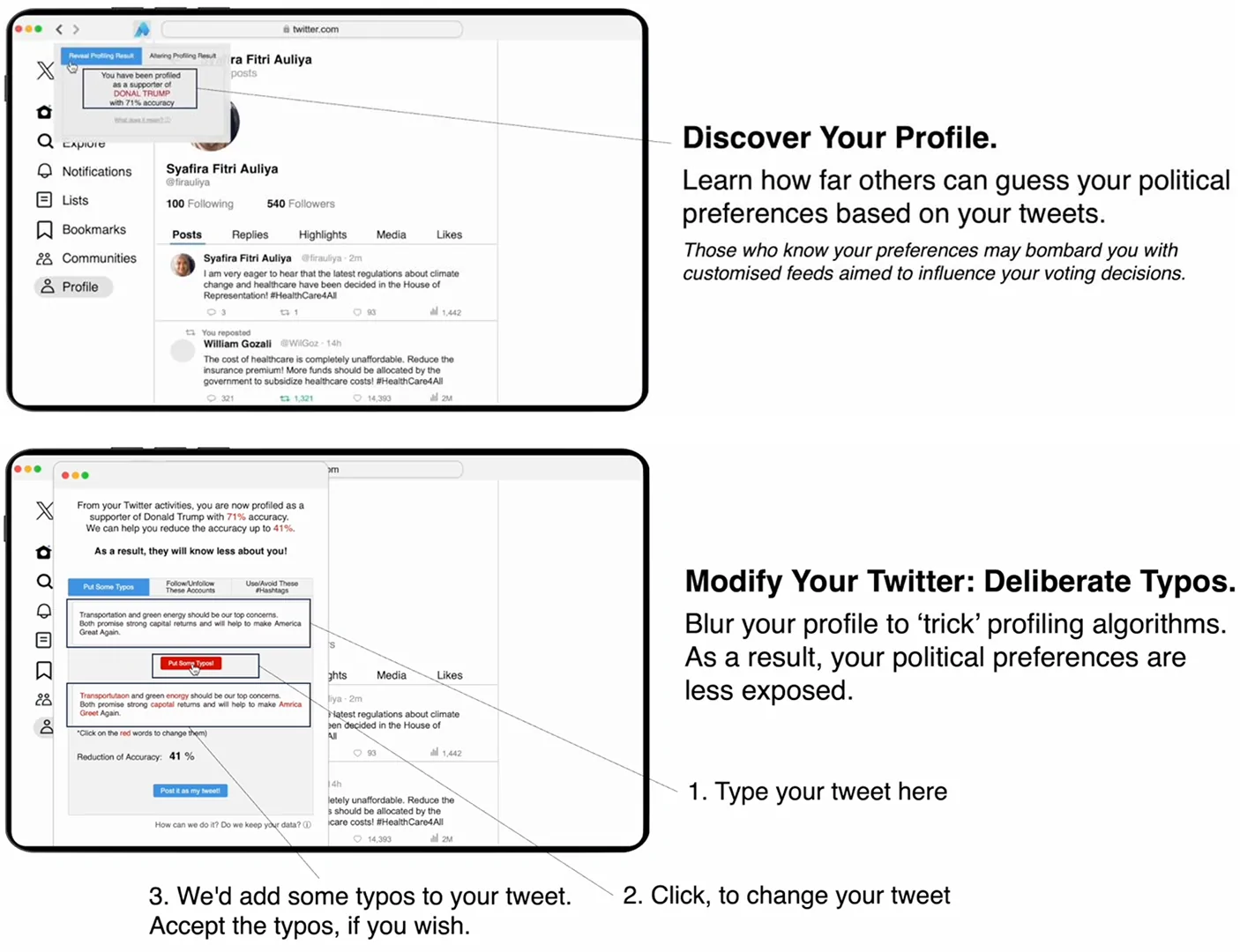
Screenshots of a (English-Version) two-minute video of APP-AI that the respondents watched

To inspire participants to imagine potential technological solutions in facilitating their deliberative decision-making in elections, we introduce a proposal called *Adversarial Privacy Preserving AI* (APP-AI). This proposal acts only as a starting point for participants to discuss and consider technological solutions in their minds without being a fixed solution. The participants were asked to watch a two-minute *video* showcasing the APP-AI we proposed, as seen in Fig. 1, followed by relevant questions. This APP-AI had two features. *Feature-1* informed users about their political preferences as profiled by AI that learned about them using their social media data. *Feature-2* modified their social media data by putting deliberative typos in future social media posts using the concept of AML, where minor alterations to data input change the AI’s output. In this case, the change in AI’s output aimed to hinder the AI profiler from making accurate users’ political profiles.

To explore the perspective of political actors, four individuals were interviewed. They were individuals running for the Indonesia 2024 elections, their campaign managers, or political/media consultants at management levels. We selected responders from diverse geographical locations to gather various points of view. The interviews centered on the political strategies they employed during the 2024 elections and the use of micro-targeting campaigns.

We conducted interviews offline and online, with offline interviews taking place in four Indonesian provinces in January 2024. The online interviews were conducted using Microsoft Teams and Zoom in 2023 and 2024. The interviews were taped and transcribed anonymously with the respondents’ written permission. The transcripts were returned to the respondents for their approval, with minor alterations made in a few cases, and one respondent asked to exclude certain parts of the interview. All interviews were conducted in the Indonesian language.

We used qualitative analytic approaches based on Reflexive Thematic Analysis (RTA) to identify, analyze, and report patterns from the semi-interview data (Braun & Clarke, 2006). In RTA, as described in Byrne (2022) and Braun and Clarke (2006), six-phase processes are used to identify recurring trends among the participants’ answers. In phase one, after transcribing the interview using the speech-to-text feature in Google Docs (as informed in the Informed Consent Form signed by all participants prior to the interview), we familiarized ourselves with the data by reading the transcript multiple times. In phase two, we created initial codes utilizing open coding. In phase three, the codes were revised as deemed appropriate by the coder and were subsequently organized into initial themes. During phase four, the themes were reviewed. The process of theme generation primarily centers on its ability to address the research questions and adhere to a set of essential criteria. These criteria include establishing a distinct boundary for the themes, ensuring the presence of sufficient meaningful data to substantiate them, and maintaining coherence throughout the analysis. We did not set a minimum mention of a theme for it to be formed; even if one respondent mentions it, but it is highly relevant to the coherence of the analysis, it may be established as a theme. The themes were defined in phase five, while the report was composed during the sixth phase. Once the process was completed, the analysis of semi-structured interviews yielded themes, as depicted in the Appendix.

We examined each interview transcript utilizing Atlas.TI 24.0.1. A predominantly inductive approach to data analysis was adopted, meaning data were open-coded to identify themes pertinent to the research question. However, deductive analysis was also employed to ensure that open-coding generated themes meaningful to the research questions. Ethical approval for this study was granted by the Human Research Ethics Committee at TU Delft, the Netherlands.

## Results

From the RTA conducted, several themes emerged. All themes contributed to the identification of design principles for the technological tools that can achieve the goal of facilitating deliberative decision-making in elections. ‘*Factors Influencing Individuals’ Election Decisions’* theme serves as a background to understand how our respondents make their election decisions, which is crucial to identify which parts of the decision-making process are being targeted by micro-targeting political campaigns. ‘*Strategies for Micro-Targeting Political Campaigns on Social Media’* examines the strategies used by political actors, informing the approaches needed to counter these strategies. ‘*Reactions’* explores people’s emotional responses to micro-targeting political campaigns and their current counter measurements, guiding technology design to align with users’ emotional states and address gaps in existing measurements. ‘*Achieving Deliberative Decision-Making in Elections’* highlights the necessities identified in our interviews that lead to the conditions required to achieve deliberative decision-making in elections. Some of the themes are divided into sub-themes, which provide a deeper understanding of the interview data, reveal contrasting dimensions, or offer more structured findings. We have translated the respondents’ quotes into English.

### Factors Influencing Individuals’ Election Decisions

We inferred several key factors potentially influencing our participants’ election decisions. These codes were grouped into two sub-themes: *Internal Factors* and *External Factors*.

*Internal Factors.* All citizen participants acknowledged that information aligning with their personal predispositions significantly impacted their election choices. The extent of this influence varies among participants, with biases seeming to play a role in this variation. For instance, a participant expressed a bias for information that strengthens his existing belief, expressing “…[we are] just wanting hearing good news that aligns with what we already believe” [P9]. Demographic biases were also noted, such as the notion that “… for Javanese people, they want someone who is modest … like how Javanese people are” [P11]. Interestingly, some participants reported being able to overcome their biases and challenge their personal predispositions. These participants tended to express a stronger sense of civic responsibility. For example, one participant, who had previously been an active member of a political party and blindly followed the party’s voting direction, said that the knowledge she gathered during her work in a government financial institution led her to prioritize objectively assessing candidates’ financial promises. Similarly, another participant felt a duty to scrutinize candidates for helping future generations experience a good government like he was benefitted during his upbringing.

*External Factors.* Our participants also emphasized the significant impact of external influences, such as social networks, endorsements from influential figures, and media coverage, in making their election choices. For instance, when participants perceived that most people in their network supported a particular candidate, they were more likely to support that candidate as well. As a participant explained: “Initially, I felt like it was not a very important issue, but it turns out that when I see many other people also have concerns about it, it will amplify what is in me.” [P3] Social media played an especially interesting role in shaping these election decisions, especially in determining whose opinions were valued. For example, young generations were influenced more by social media influencers than by traditional experts, as expressed by participants: “young people even trust [social media] influencers more than professors… because they have been following these influencers for a long time, and they see them as good role models.” [P16] and “I usually get quite influenced if many accounts that I trust are posting the same thing.” [P7] In addition to these conversational-level endorsements, another significant influence on Indonesian voters was direct compensation. Participants agreed that monetary benefits given to prospective voters, a practice known as patronage, significantly affect people’s voting.

### Strategies for Micro-Targeting Political Campaigns on Social Media

We asked our political actor participants about their micro-targeting strategy in generating political campaigns, especially on social media. Two sub-themes were identified: *Data Gathering* and *Delivery Strategies*.

*Data Gathering.* Data gathering always initiates campaign strategy development. In traditional campaign teams, political actor participants relied on personal networks and campaign teams (referred to as ‘tim sukses’) to gather information. However, participants proficient in technology also employed computational data analytics methods, facilitating the simulation of diverse strategies using data. This data was obtained from external entities and social media platforms, allowing these participants to optimize resource allocation and target individuals susceptible to specific campaign materials. This analysis was conducted either internally by in-house digital teams or outsourced to external entities. Nonetheless, none of our participants engaged in large-scale personal data gathering and analysis. While Personal Identifiable Information (PII) is often illegally available in Indonesia due to weak law enforcement and a low understanding of privacy or data regulation, participants who had access to these data said that they did not leverage such data for sophisticated micro-targeting campaign strategies. Although participants acknowledged that processing PII could enhance personalization and voter impact, the substantial expenses involved in data collection and analysis provide a significant obstacle, according to them.

*Delivery Strategies.* After gathering and analyzing data, the campaign materials were generated and delivered. Here, we noticed that the level of micro-targeting in Indonesia’s 2024 elections differed from cases such as the Cambridge Analytica scandal. In that scandal, the whistleblower explained that they knew “each and every voter and [we] may be whispering one thing to this voter and another thing to another voter.” (Wylie, 2018), that indicates the delivery of personalized materials at the individual level. In contrast, our interviewees reported that the micro-targeting in Indonesia’s 2024 election was conducted at a more aggregated level.

Of the four participants, one with less technological usage tailored their micro-targeting campaign materials to diverse communities based on data from their networks and ‘tim sukses.’ For instance, using the data, participants identified that in less prosperous areas, potential voters were inclined towards discussions on fundamental necessities like guaranteed basic education and employment availability, while in wealthier residential complexes, infrastructure was a predominant topic. The other three participants used more technological elements in their campaigns and more advanced methods for delivering micro-targeting campaigns. Following the analysis of aggregated social media data, campaign materials were delivered to the respective aggregated targeted groups. For example, participants created campaign videos featuring traditional clothing and symbols of specific ethnic and religious groups, which were then disseminated via targeted social media advertisements, with recipients filtered the recipients based on the geographical area of the constituents. In fact, a participant recognized that their campaign strategy could be further enhanced by using additional filtering that targeted audiences based on the additional combination of religious and ethnic identity – a unique trait of this participant. Yet, this participant lacked access to more advanced technology that offers such specific filtering options and relied on offline methods to target prospective voters who valued these identities.

These participants also highlighted the importance of adapting strategies to different platforms. Twitter, for example, was used by more mature voters who wanted to access diverse political perspectives via text-based materials. Twitter users tend to be very critical of the content of the information given and the credibility of those who spread the information. Meanwhile, younger participants, including first-timer voters, exhibited less familiarity with Twitter and displayed a stronger engagement toward platforms such as TikTok and Instagram. The influence mechanisms on these two platforms also differ from those on Twitter. Campaign materials on TikTok, in particular, are more emotionally connected to the candidates.

### Reactions

We asked our citizen participants about their perspective on being the target of micro-targeting political campaigns. We categorized the coding derived from these perspectives into two sub-themes: *Emotions* and *Countermeasumenets*.

*Emotions.* All citizen participants were aware that their social media content was tailored for them, though they were unsure about the extent of data analysis involved. They expressed mixed feelings regarding the micro-targeting political campaigns they received. Some participants accepted it as part of the platforms’ business process. They understood that social media platforms function based on a profit-oriented model, and enabling advertisers to target consumers is one of the methods employed to sustain the business. A participant argued: “We use social media for free; users don’t need to pay subscription fees. So, if there’s no information or something that can be sold from there, the platform won’t survive. [I do not mind] as long as the use of that information remains at the appropriate level.” [P3]. Nonetheless, some participants reacted to micro-targeting political campaigns differently than in other non-political contexts. For example, a participant expressed, “If … a company is trying to find out my shoe preferences, clothes, or bags, I might not mind. In fact, I would be happy if I were given shoe or fashion recommendations that I like. But, when someone tries to read my political preferences, … I am uncomfortable with that”. [P7].

*Countermeasumenets.* When participants feel unease with how social media platforms use their data, either because of concerns regarding privacy, filter bubbles, a hindrance in decision-making, or dissemination of unverified content, they develop some mechanisms to make them more at ease. For example, some participants adjusted their online behavior to obfuscate their political preferences to prevent others from knowing and tailoring micro-targeting materials for them. A participant using Twitter expressed that she usually manually put different keywords on the search bar to make the Twitter algorithms confusing her political preference, stated: “When the [feed] consistently leans towards one side only, no variety, nothing else emerges, I try to find something different by typing [different keywords]. For instance, if the feed is [only] about Prabowo, I will type [keywords] about Anies … Because I read Twitter every day, I can feel [when the feed has already been saturated]” [P6]. Another participant used Tiktok’s refresh feature^2^, which allows her to reset the ‘For You’ feed and offers more varied content. Nonetheless, she acknowledged that in addition to not all Tiktok user’s participants knowing about this feature, this feature will not change the personalized profile gathered for ads and other feeds.

### Achieving Deliberative Decision-Making in Elections

In our semi-structured interviews, we presented a prototype (APP-AI) to trigger participants’ imagination about the potential of technologies to help facilitate their election deliberation, such as by avoiding being accurately profiled on social media. All participants were excited about *Feature-1*, which allowed them to be informed on how AI inferred their political preferences. Participants felt that if AI could guess their political preferences using their social media data, they would feel unfree to use social media. However, *Feature-2*, which introduced typos to blur social media data, received mixed reactions. They appreciated this feature, especially because it allowed them to have control over which words to alter with typos, with the tool informing them of the consequences of each decision. Yet, the acceptance of typos varies. Some participants totally welcomed the use of the typos. They had some hints that typos could fool social media algorithms because of widespread support for Palestine among Indonesians. Many of their social media friends already used terms like “G4Z4” or posted pictures of iconic watermelons when discussing this topic, hoping to escape the censorship by (biased) social media platforms’ algorithms^3^. However, some participants who initially accepted the use of typos to protect themselves from political profiling changed their stance after follow-up questions asking them to imagine a person who had frequent typos in their social media posts. They realized that their perception of this imaginary individual’s credibility decreased. Consequently, these participants reconsidered using typos to avoid being perceived negatively by others on social media.

Additionally, participants with expertise in Natural Language Processing (NLP) also doubted the effectiveness of typos in ‘fooling’ AI. A participant explained: “How easy is it to bypass typos? Very easy. Current AI models don’t look at individual words anymore; they analyze them at the sub-word level … For example, the word ‘pertahana’ can be broken down into ‘per-ta-ha-na’. This breakdown makes the model less vulnerable to typos. So if you have a typo like ‘per-ta-ha-ta’, only one subword is incorrect, and the rest is still recognized correctly. Machine learning models are quite robust because they don’t just look at words individually but consider the context. Therefore, even with a subword typo, the context helps maintain a robust prediction.” [P15]. Instead of typos that were very easily bypassed, he suggested some alternatives to alter social media data, such as satire, humor, paraphrasing, and negation, which current AI models still struggle to understand.

Participants with expertise in computer science also suggested additional details for *Feature*-*2*. They suggested that the result of *Feature-2* should not only reduce the accuracy of AI profiling users’ political preferences based on users’ acceptance or rejection of suggested alterations. But, instead, APP-AI should aim to neutralize users’ political profiles. So, APP-AI should modify users’ profiles to appear as equally supportive of all candidates. This neutralization would hinder micro-targeting political campaigns from accurately detecting and strengthening users’ preexisting echo chambers.

We also noticed that the participants, according to their self-reporting, expressed different levels of deliberateness in their election decisions. As proposed by Auliya et al., (2024) we use the term ‘deliberative decision-making in elections’ as a means for citizens to make independent, reflective decisions. Exploring the participants who believed that they have a higher level of deliberation (e.g., by stating that “I am quite rational [in deciding my voting decisions]” [P1] and “[I think critically] and reflect more” [P9]”), we categorized their testimonies into several key characteristics that seem to determine the degree of deliberation in election decisions:

- *Reflecting.* The respondents recognized their personal predispositions and reflected on these predispositions. Whether their voting decisions eventually aligned with their initial predispositions or not, the main takeaway was the recognition and reflection on these predispositions prior to making election decisions. For instance, a participant shared how she was more reflective in this election because she discovered her personal predispositions after encountering an issue disturbing her [P7]. Similarly, another participant emphasized the importance of critically evaluating candidates based on her predisposition: “The information we receive is never completely true. … [we] really [need to] evaluate whether [candidate’s] characteristics are … align with [our] value.” [P4] This reflection acts as a catalyst, encouraging participants to critically assess the information they encountered.
- *Awareness of Political Manipulation on Social Media.* The respondents were generally aware that political actors use social media as part of their campaign strategies. Although they were unsure about the exact use of social media data to construct targeted political campaigns, they acknowledged the potential for political manipulations. This awareness contributed to their ability to make deliberative decisions, as it made them more cautious in digesting information online. For example, a respondent stated, “If [political actors] try to persuade people, even using hoaxes or illegal and immoral means, … it is their business. My domain is to sift through that persuasion, filter the advertisements, and choose the information. So, I do not feel restricted [in making deliberative choices]“ [P1]. This quote illustrates that the respondent was aware that he needed to maintain control over his decisions since he knew that he was surrounded by potential manipulations.
- *Thinking Critically*. Recognizing the potential for information to be manipulative, several respondents critically assessed what they encountered. Some participants argued that having a solid knowledge base of diverse issues might facilitate this critical approach, stating, “If we have a solid knowledge base, we can make decisions and filter how we respond to a particular issue. …Without a strong knowledge base, people can easily be influenced.” [P3]. Nonetheless, other respondents observed that knowledge or even education does not always correlate directly with critical thinking. For instance, they mentioned that their highly educated friends still blindly followed the voting direction from external influences without much critical thought. A respondent connected this to his assumption that, for these friends, politics is less relevant to their lives. He assumed, “[my friends] are likely more focused on doing their studies and work … They also do not feel that politics will have much impact on their lives”. [P9] Therefore, having a personal motivation and the belief that one’s vote matters appear important in enhancing critical thinking in election decisions.
- *Seeking Diverse Information*. Some respondents actively sought out diverse information about all candidates, believing this approach was essential for making objective and critical voting decisions. As highlighted by some participants: “to avoid getting too focused on that same topic, I also have to be creative in managing my social media so that my bubble doesn’t become too concentrated.“ [P6] and “I follow all politicians from various political and ideological spectrums. … So, I get exposed to different political opinions. … this helps reduce the risk of really falling into that echo chamber.” [P9] Nonetheless, obtaining diverse information alone was *not* always sufficient to reflect or critically assess the already existing political views. A participant who worked in echo chamber research mentioned: “In my model, there are people who … are not open-minded. They have access to a lot of information. … They are exposed to all kinds of information. [But], they [just] choose to stick to their point of view” [P10]. This highlighted the limitations of diverse information in directly connecting and facilitating deliberative decision-making. Similar hints were also given by other respondents. These participants, for example, were only able to welcome alternative perceptions after being encouraged by people they valued. For instance, [P6] previously only read political materials that aligned with her views. But, she changed her behavior after hearing a family member’s alternative political perspective. Similarly, [P16] reassessed his information sources after taking a course on media framing, and his final push was interacting with respected seniors who provided different political perspectives. Their testimonies suggest that while seeking diverse information can support deliberation, being open to and reflecting on alternative information often requires personal emotional connections with the sources.

## Discussion

This section offers reflections on the findings presented in the previous section. Section  (“Interpreting the Finding: Implications for Deliberative Decision-Making in Elections”) interpretes the findings and discusses their implications for deliberative decision-making in elections, closed by our proposed design principles and practical recommendations. Section  (“Limitations of the Study”) presents the limitations of the study.

### Interpreting the Finding: Implications for Deliberative Decision-Making in Elections

Our interview findings suggest that people’s susceptibilities are shown, such as through their past posts, likes, or the accounts they follow, and political actors can target them with campaign propaganda aligned with these topics. Given that every individual carries experiences that can be brought to the fore during a period of intensive influence, these campaign propagandas become catalysts that bring the predispositions to the level of visibility and expression until they manifest as a vote (Lazarsfeld et al., 1993). Therefore, micro-targeting political campaigns that target individuals’ predispositions leverage the influences of campaigns on citizens’ voting decisions much more than generic campaigns.

Empirically, political actor participants in our study provide a further understanding of how micro-targeting political campaigns work. These campaigns often target prospective voters by leveraging aggregated data of groups of voters, such as geographical groups, ages, gender, ethnicity, and topic of interest. While this practice was not yet widely used in Indonesia’s 2024 elections, in more technologically advanced campaigns, the use of AI can use more detailed data about people to enable more detailed targeting. Imagine this hypothetical example: a political campaign initially had one big group of 10,000 prospective voters based on city and age. By adding more information about people’s religion and ethnic data, this one group can be refined to 20 groups of 500 prospective voters. These 20 groups will be given more specific personalized campaign messages that contain much stronger campaign materials about religion and ethnicity for each group. Since the ability to identify religion and ethnicity using social media is already a topic of research (Chaturvedi & Chaturvedi, 2024; Golder et al., 2022), the above hypothetical scenario may become a reality in the foreseeable future. In addition, AI technologies that generate tailored video and text content for specific audiences could make the creation of personalized campaign materials even more accessible. Political actors in our study, who expressed a desire to implement advanced micro-targeting strategies but lacked sufficient technological resources, may find these AI-driven tools a game-changer. By providing deeper insights into prospective voters and enabling the generation of tailored campaign materials, these AI technologies could significantly enhance the scope and penetration of micro-targeting political campaigns in the future.

Meanwhile, based on the interview data, we observed that although our citizen participants are already aware of the existence of micro-targeting political campaigns on their social media, not all of them realize the negative impacts of such campaigns on deliberative decision-making in elections. The convenience they experience online often makes them allow the use or access to their data to services that could exploit their political preference and facilitate micro-targeting campaigns. This suggests the importance of educating people about the dangers of micro-targeting political campaigns. However, as Auliya et al. (2024) argue, education alone is insufficient to ensure deliberative decisions in this age of AI. As AI develops more, it may generate political influences that are subtle, massive, and less fully understood by most people. Even well-educated people cannot reasonably remain vigilant against every influence all day. To address this, they propose the use of AML to enhance privacy on social media, so that citizens’ susceptibilities can be hidden from profiler AI used by political actors (Auliya et al., 2024). Nonetheless, our findings suggested that while AML techniques show promise and, if technologically reliable and less obvious, might be welcomed in protecting privacy on social media, they alone are *not* sufficient to achieve deliberative decision-making in elections.

From a normative perspective, enhancing privacy on social media indeed offers better data protection. Leaving people alone by preventing people’s political preferences from being known might reduce the effectiveness of micro-targeting political campaigns since the campaign-makers know less about people’s susceptibilities. With less disturbance from micro-targeting political campaigns that exploit their susceptibilities, people might have more room to reflect on their choices before making decisions in elections and obtain a broader range of political information before voting. Nonetheless, our findings also indicate that neither having more reflection room nor more exposure to diverse political polarizations guarantees better reflection on the information gained.

Merely exposing people to different viewpoints sometimes backfires. Chris Bail, in his book ‘Breaking the Social Media Prism,’ found that the participants who were exposed to opposing viewpoints often became more polarized instead of moderating their views. When his participants stepped outside their bubble and saw their side being attacked, they tended to defend themselves, sharpening the “us and them” mentality (Bail, 2021). According to Bail, a key way to reduce polarization is to reshape perceptions of opposing polarization and encourage more empathic discussions. Similarly, Ngunyen (2020) distinguishes between an “epistemic bubble” and an “echo chamber”, arguing that an epistemic bubble is a condition when one is unintentionally left out of some information but, in contrast, an echo chamber actively excludes and discredits other opinions. Escaping an epistemic bubble can be achieved by mere exposure to previously unobtained information, but escaping an echo chamber requires a radical rebooting of one’s belief systems. This involves being aware that they are in the echo chamber’s grip and forming a trusting relationship with outsiders (Nguyen, 2020). Settle further argues that the amount of perceived polarization is larger among those with higher social distance (Settle, 2018), indicating the importance of close social connections in breaking people’s echo chambers. All these scholars’ arguments highlight the necessity of social and emotional connection predeceasing independent and reflective voting decisions. Our results also align with these perspectives. As revealed in Section  “Results”, two participants who initially held strong political opinions driven by external influences began to reflect on alternative perspectives only after encouragement from trusted individuals within their social network. This suggests the importance of emotional connection in catalyzing the reflection process.

**Fig. 2:**
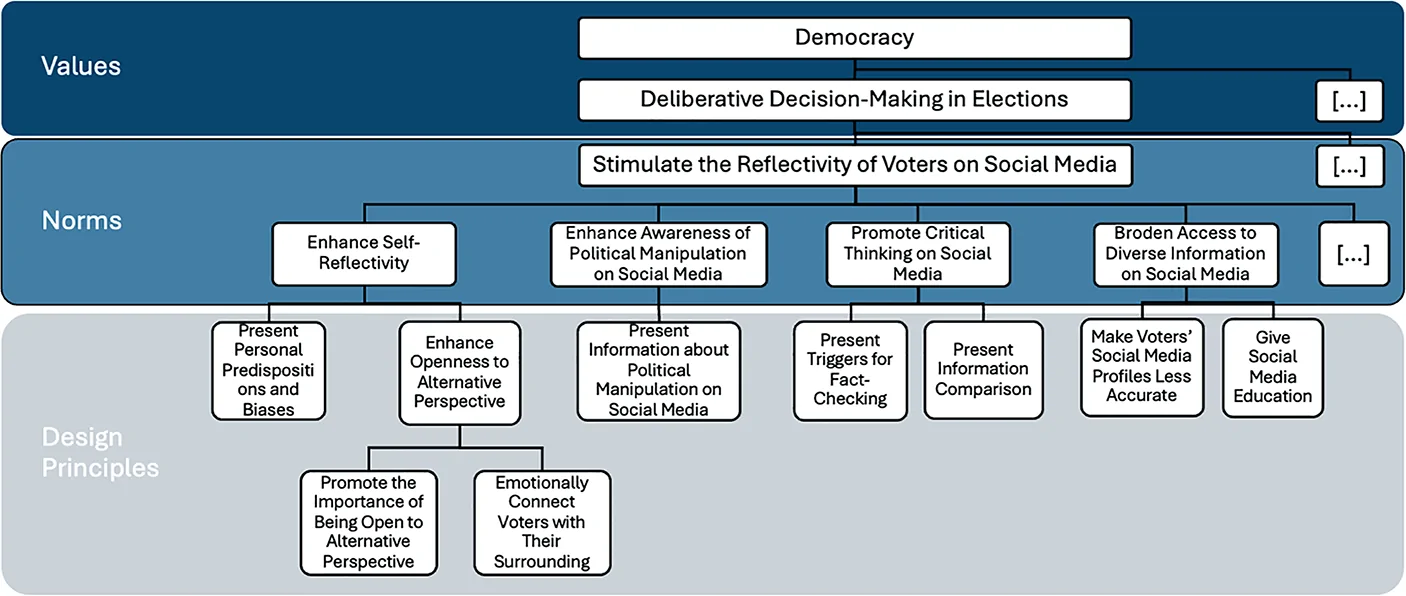
Value hierarchy to enhance democracy thought facilitating deliberative decision-making in elections

Thus, these empirical observations and normative thought motivate the following design principles. We conclude that deliberative decision-making in elections can be enhanced when people (1) recognize and reflect on their personal predispositions and biases, (2) are aware of political manipulation, such as on social media, (3) think critically, (4) seek diverse information, and (5) are open to alternative perspectives. Based on this summary, we apply the value hierarchy suggested by Van De Poel (2013) to specify norms and design principles for technological solutions aimed at facilitating deliberative decision-making in elections, as seen in Fig. 2.

As seen in Fig. 2, ‘*deliberative decision-making in elections’* can be seen as an instrumental value supporting the intrinsic value of ‘*democracy’*. In this value hierarchy, ‘*stimulating the reflectivity of voters on social media’* is presented as the high-level norm, while the five qualities outlined previously are presented as lower-level norms. More precisely, the first two qualities are combined since they are strongly related, awareness of political manipulations is presented as a standalone norm, and the last two qualities become each a norm.

As Van de Poel has stated, this translation process is value-laden, and different disciplines might offer different ways to specify a value (Van De Poel, 2013). Our design principles are derived from our empirical study and the previous discussion in this section. Based on these, we offer some design principles to achieve each norm. These design principles act as guidelines for technological solutions aimed at achieving each norm, which in turn achieves the overall purpose of enhancing democracy.

To achieve the norm of ‘*enhance self-reflectivity*,’ we suggest two design principles. *First*, presenting users with insights into their own personal predispositions and biases, which can trigger them to consider how these predispositions and biases shape their political opinions. A relevant example from Indonesia’s 2024 elections is the website ‘Bijak Memilih’ that offers two quizzes: one identifies users’ key issues of interest based on their answers, and the other compares users’ policy choices with political parties’ actions, indicating alignment (e.g., “you are 80% similar with Party A”). Users can share their results on social media. From reactions posted online, many people were surprised by how closely they aligned with parties they previously opposed. This led them to reconsider and reflect more deeply on their political preferences. *Second*, enhancing users’ openness to alternative perspectives involves endorsing their importance and fostering people’s trust in their surroundings. A paper by Maria & Roeser (n.d.) suggests notifications like “imagine others as like your family members” to trigger people to treat others online with more empathy. Another approach is to highlight overlapping perceptions between users and their surroundings, helping users realize that those around them share some common ground, thus fostering trust and openness.

To fulfill the norm of ‘*enhance awareness of political manipulation on social media*,*’* we suggest presenting information about these manipulations, their dangers, and real-life examples. The *Feature-1* of APP-AI is one example of the implementations that may fulfill the design principles by revealing how Profiler AI generates users’ political profiles. This feature allows voters to become aware of the influence of targeted campaigns and reflect on how external factors may shape their perceptions.

To ‘*promote critical thinking on social media’*, we suggest tools that encourage fact-checking. The encouragements can be in the form of education about its importance, constant pop-up notifications, or even warnings if the users are only constantly exposed to misinformation. Leveling this up, technologies can also be used to compare information from multiple perspectives.

Lastly, to ‘*broaden access to diverse information on social media*,*’* enhancing privacy could be the key. As political actors often rely on data accessed through social media to generate their micro-targeting political campaigns, limiting the amount of users’ essential information available to these political actors could reduce the relevance and effectiveness of such campaigns. As Auliya et al. (2024) argued, while privacy enhancement will not eliminate micro-targeting political campaigns, these campaigns will have less impact on voters if the campaigns lack relevance to voters’ susceptibility, leaving voters with more space to reflect before making their deliberative voting decisions (Auliya et al., 2024). Nonetheless, as revealed by our participants, this privacy-enhancement technology should have some key features, such as technological reliability to build users’ trust, user control of their actions and data, and transparency so that users will be fully informed about the consequences of their choices. Educating users on the importance of diverse information is also necessary.

In sum, these principles suggest that achieving certain values like deliberative decision-making in elections rarely comes from a single silver bullet solution. Instead, a combination of several interrelated approaches is necessary. While we do not claim that the proposed design principles and corresponding technological solutions will directly ensure deliberative decision-making, they may help to offer concrete, actionable insights that can guide future research and technological developments to achieve the goal of election deliberation and enhance democracy. Importantly, technical solutions must be embedded within a broader ethical and societal framework to be effective (Chen et al., 2023).

Building on these principles, several practical recommendations can be articulated for key stakeholders. *First*, for the data protection regulators. Data subjects should be explicitly informed when automated systems infer their political preferences for political messaging. Such information should include the general logic of the profiling methods and the data involved. For instance, in the Indonesian context, the Personal Data Protection Law (UU No. 27/2022) already addresses profiling that can affect individuals (Chap. 10 – “Personal Data Subjects have the right to object to decisions based solely on automated processing, including profiling, that produce […] significantly impact the Personal Data Subject”) and establishes the right to be informed about the use of personal data (Chap. 5 – “Personal Data Subjects are entitled to obtain information about […] the purpose of requesting and using Personal Data”). However, the information about being targeted in political micro-targeting and the data used for it are not yet explicitly covered. Derived regulations should clarify these areas to address the gaps. *Second*, for election commissions, additional regulatory requirements should be introduced to mandate disclosure of micro-targeting practices in political campaigns. Campaign actors should be required to report whether and how micro-targeting is used, including why particular messages are shown, which data informs targeting, and who funds the campaigns. In Indonesia, such requirements could be incorporated into existing campaign regulations issued by the Komisi Pemilihan Umum (KPU), for example, within provisions governing campaign advertising (e.g., Paragraf 4 Iklan Kampanye under UU No. 7/2017). Currently, that regulation emphasizes fairness, balance, and equal access to campaigns, but does not yet account for the differential effects and imbalances introduced by algorithmic micro-targeting. *Third*, for technological developers and political campaign consultants, the development of micro-targeting technologies should prioritize social legibility. Yet, above all, these recommendations apply to ideal conditions. As stated in the Introduction, the proposal to use AML-based privacy-enhancing technology reflects a non-ideal condition in which citizens may need to protect themselves. While self-protection, as discussed in this paper, is insufficient on its own to achieve election deliberation, it remains preferable to inaction and should be understood as one component within a broader and multidimensional framework.

### Limitations of the Study

We acknowledge that this study has several limitations. *First*, our study is grounded in Indonesia’s 2024 election and focuses on a single social media platform. Our aim therefore is not statistical generalization. The challenges posed by microtargeting in political campaigns on social media are not unique to a single country or platform and have been documented across diverse electoral settings. But at the same time, the contextualization of the problem and the effectiveness of the proposed interventions indeed depend on context-dependent variables, such as citizens’ trust in government, prior viral political controversies, and the level of technological penetration in the country. For example, in countries with low institutional trust and weak regulatory enforcement, presenting information about political manipulation on social media may play a central role, enabling people to be more ready to protect themselves. Consequently, while the proposed design principles are intended to be broadly applicable, their concrete implementation should also be adapted to local conditions. Future research could strengthen these findings by replicating the study across multiple countries and platforms to identify which principles are stable and which are context-dependent.

*Second*, this study relies on qualitative methods to explore stakeholders’ perspectives. To avoid overstating claims, the proposed design principles should be treated as principles to be further evaluated and validated. Future studies can strengthen the proposed principles by complementing them with quantitative evaluation. At least two quantitative lenses can be adopted: (1) technical effectiveness, for example, by experimentally measuring changes in the predictive accuracy of political profiling models before and after the application of the AML-based privacy-enhancing technology, and (2) user-level perspectives, for instance, by assessing and testing whether the interventions help users to realize the relevant values e.g., democratic deliberation.

*Third*, the key limitation of AML lies in the evolution of countermeasured AI systems. As noted in prior conceptual work preceding this study by Auliya et al. (2024), the proposal of using AML to counterattack microtargeting political campaigns based on AI resembles a cat-and-mouse game, in which profiling AI systems may adapt and change their algorithms after learning about the existence of AML. But, despite the limitation, such approaches may still serve as one potential remedy to the current state of affairs. Their effectiveness should therefore be periodically reevaluated and updated in response to evolving technological conditions.

## Conclusion

This paper presents an explorative empirical study to explore the possible design principles to achieve the value of deliberative decision-making in elections and enhance democracy. Building on the conceptualization of Auliya et al. (2024), we initially assumed that privacy-enhancing AI, facilitated by AML, could enhance deliberative decision-making in elections. However, our findings reveal that the realities of the election choices are often complex and messy. While enhancing privacy on social media through AML can limit the impact of micro-targeting political campaigns, privacy alone is insufficient for achieving deliberative decision-making. The combination of several interrelated approaches is necessary, i.e., to help discover personal predispositions and biases, compare the information with personal predispositions, help emotionally connect voters with surroundings, and help provide diverse information comparison. These suggested approaches became our proposed design principles in this paper.

As part of a larger VSD study, these design requirements offer initial guidance for designers working to develop technological solutions aimed at facilitating election deliberation. Yet, our findings are only based on respondents from a single country, and potential solutions presented were focused on specific social media platforms. Expanding the study across various countries and platforms in future larger-scale studies might reveal additional context-dependent insights not captured here. If so, given VSD’s iterative development, the design requirements identified in this paper shall be refined and revisited if any value conflicts or trade-offs emerge in subsequent stages. Therefore, following investigations, e.g., technical investigation or larger-scale empirical investigation, might reveal the potential of these design requirements suggested in facilitating deliberative decision-making in diverse contexts.

## Data Availability

Not applicable.

## Footnotes

1 In 2018, the Chief Technology Officer of Facebook posted Facebook’s plans to restrict data access on Facebook as part of their responses to the Cambridge Analytica scandal. The plans included that they “no longer allow apps to ask for access to personal information such as … political view…” (retrieved from https://about.fb.com/news/2018/04/restricting-data-access/ on March 12, 2024). They also discontinued the ‘Partner Categories’ feature, which previously allowed third parties to match information about a user’s offline data with their Facebook activity. (retrieved from https://about.fb.com/news/h/shutting-down-partner-categories/ on March 12, 2024).

2 See https://support.tiktok.com/en/account-and-privacy/account-privacy-settings/refresh-your-for-you-feed.

3 See https://www.theguardian.com/technology/2023/dec/21/meta-facebook-instagram-pro-palestine-censorship-human-rights-watch-report.
